# Efficacy of probiotic adjuvant therapy for irritable bowel syndrome in children: A systematic review and meta-analysis

**DOI:** 10.1371/journal.pone.0255160

**Published:** 2021-08-06

**Authors:** Hua-Lan Xu, Li-Li Zou, Mao-bing Chen, Hua Wang, Wen-Ming Shen, Qi-Han Zheng, Wei-Yan Cui

**Affiliations:** 1 Department of ICU, Wujin People Hospital Affiliated with Jiangsu University, and the Wujin Clinical College of Xuzhou Medical University, Changzhou Jiangsu, P. R. China; 2 Department of Nursing, Wujin People Hospital Affiliated with Jiangsu University, and the Wujin Clinical College of Xuzhou Medical University, Changzhou Jiangsu, P. R. China; 3 Department of Emergency, Wujin People Hospital Affiliated with Jiangsu University, and the Wujin Clinical College of Xuzhou Medical University, Changzhou Jiangsu, P. R. China; Osaka City University Graduate School of Medicine, JAPAN

## Abstract

**Objective:**

Irritable bowel syndrome (IBS) affects children’s quality of life and learning. The purpose of this research was to systematically evaluate the efficacy of probiotic adjuvant therapy for IBS in children.

**Methods:**

The Web of Science, PubMed, Cochrane Library, EMBASE and Clinical Trials databases were electronically searched for randomized controlled trials (RCTs) published prior to January 2021 exploring the use of probiotic adjuvant therapy for IBS in children. Strict screening and quality evaluations of the eligible articles were performed independently by 2 researchers. Outcome indexes were extracted, and a meta-analysis of the data was performed using RevMan 5.4.1 and STATA 16 software. Finally, the risk of bias in the included studies was assessed with the RCT bias risk assessment tool recommended in the Cochrane Handbook for Systematic Reviews of Interventions (5.1.0).

**Results:**

A total of nine RCTs were included. In children, probiotics significantly reduced the abdominal pain score (I^2^ = 95%, SMD = -1.15, 95% (-2.05, -0.24), P = 0.01) and Subject’s Global Assessment of Relief (SGARC) score (I^2^ = 95%, MD = -3.84, 95% (-6.49, -1.20), P = 0.004), increased the rate of abdominal pain treatment success (I^2^ = 0%, RR = 3.44, 95% (1.73, 6.87), P = 0.0005) and abdominal pain relief (I^2^ = 40%, RR = 1.48, 95% (0.96, 2.28), P = 0.08), and reduced the frequency of abdominal pain (I^2^ = 2%, MD = -0.82, 95% (-1.57, -0.07), P = 0.03). However, we found that it might not be possible to relieve abdominal pain by increasing the daily intake of probiotics.

**Conclusions:**

Probiotics are effective at treating abdominal pain caused by IBS in children, however, there was no significant correlation between abdominal pain and the amount of probiotics ingested. More attention should be given to IBS in children, and a standardized evaluation should be adopted.

## Introduction

Irritable bowel syndrome (IBS) is a type of functional gastrointestinal disorder (FGID) and is the most commonly diagnosed gastrointestinal disorder in the USA [1]. In the United States, the prevalence of IBS is approximately 16% (male: 10.5%, female: 19.2%) [2]. The symptoms of IBS are frequent and unexplained and mainly include abdominal pain, bloating and changes in bowel movements [3]. The incidence of IBS is higher in individuals under 45 years of age. The incidence of IBS is high and may affect 20% of school-age children worldwide [4]. This disease could significantly affect the quality of life and the progress of learning in childhood [5]. There are several treatment options for IBS, including diet control, medication, psychological intervention, and other adjuvant treatments; however, most of these treatments have not been tested in high-quality studies [6,7]. Moreover, diagnosis and prognosis remain very challenging.

The pathogenesis of IBS might be related to dysbiosis of the gut microbiota in the intestine [8], and supplementation with probiotics is one of the methods of treating IBS. A healthy gut microbiota plays an important role in the intestinal tract [8,9]. There are two main methods of improving the gut microbiota, namely, fecal microbiota transplantation (FMT) and supplementation with probiotics. FMT might be an effective treatment for some intestinal diseases, including *Clostridium difficile infection* [10,11], *colitis* [12], *constipation* [13], and *IBS* [14], but the method of administration is often not feasible for children. Supplementation with probiotics is another method of improving the gut microbiota. In trials with adults, probiotics have been shown to have efficacy for the treatment of IBS, and this efficacy was found to be positively correlated with the dose [15]. However, there is still no systematic review on the efficacy of probiotics in children.

In the pre-experiment of our study, we found that in trials on children, their intervention methods were similar, but their outcome indicators were not uniform. This made it difficult to perform a meta-analysis. This study tried to overcome these difficulties and systematically evaluated the efficacy of probiotics in children with IBS.

## Materials and methods

### Design and registration

This protocol has been registered on the international prospective register of systematic reviews (PROSPERO), registration number: CRD42021229816. No ethical approval was required since this study used data that were already in the public domain [16]. (URL: https://www.crd.york.ac.uk/PROSPERO).

### Study selection

#### Study type

All the trials included in this study were randomized controlled trials (RCTs).

#### Study object

Patients aged between 4 and 18 years with IBS met the diagnostic criteria (Rome II~IV), and those with other acute or chronic diseases were excluded. The inclusion criteria varied across studies according to the study objectives.

#### Intervention

Patients with a clear diagnosis were randomly divided into two groups: the intervention group (probiotics group) and the control group (placebo group). The children were required to take probiotics or a placebo regularly and record the time they were taken.

#### Outcome indicators

The following outcomes were assessed and compared with the effects of the placebo:

1. Abdominal pain score
2. Subject’s Global Assessment of Relief (SGARC) score
3. Abdominal pain treatment success
4. Abdominal pain relief
5. Frequency of abdominal pain
6. Rate of bloating after treatment
7. Standard abdominal pain and daily intake of probiotics

First, there are several methods to score abdominal pain, and this study did not limit the scoring methods. For the included studies, the larger the score, the more severe the pain; otherwise, we used the full score minus the score to be included in the analysis.

SGARC is the weighted sum of the scores of 5 subitems. SGARC is a more systematic method to assess the severity of children’s IBS.

Finally, abdominal pain treatment success was defined as the absence of pain. After excluding abdominal pain treatment success, we defined other improvements as abdominal pain relief.

To observe the relationship between daily intake of probiotics and abdominal pain, we defined standard abdominal pain (SAP) as (the mean difference of abdominal pain score) ÷ (total score of abdominal pain). We used this method to standardize abdominal pain. Additionally, we analyzed the relationship between the daily intake of probiotics and SAP.

#### Exclusion criteria

Studies whose data could not be extracted or utilized; studies on animal experiments; literature reviews, reports with duplicate data, studies with defects in research design or of poor quality, studies with incomplete data or unclear outcome effects, studies that did not undergo peer review, etc. were excluded.

### Data sources and searches

**Fig 1:**
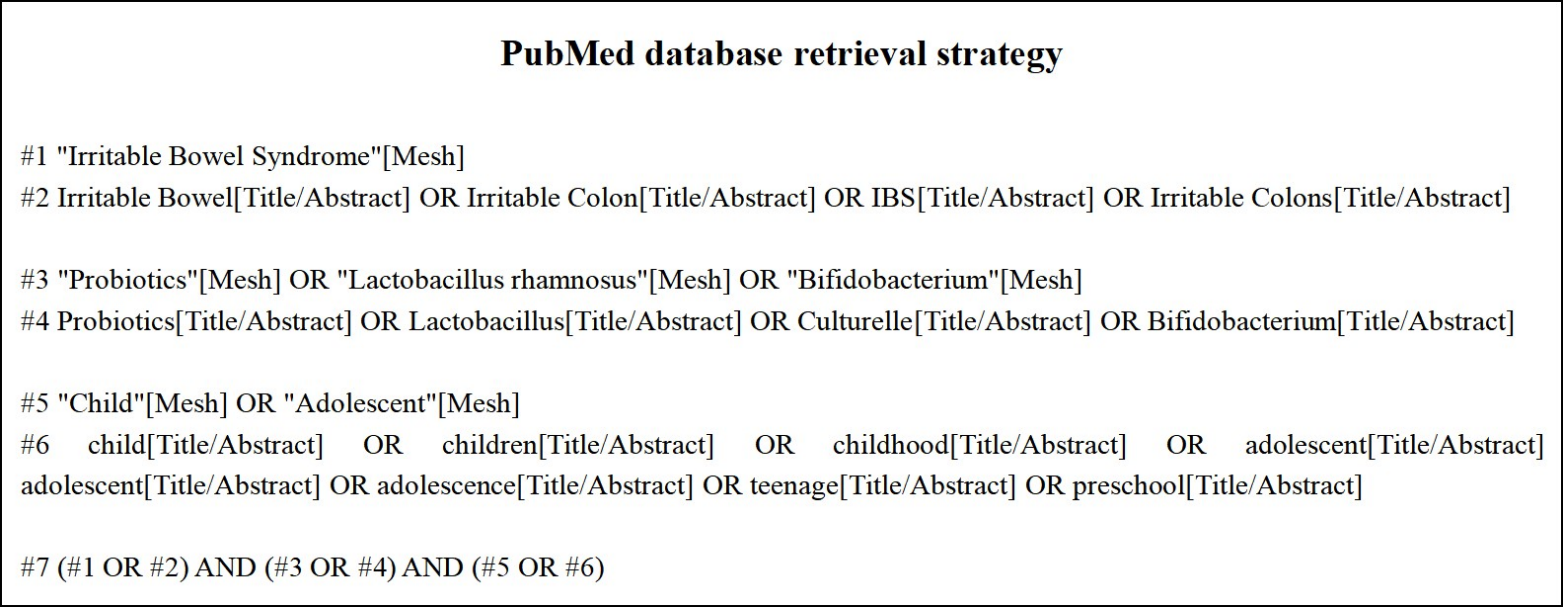
PubMed database retrieval strategy.

We searched for articles published in English prior to Jan 2021 in the following databases: Web of Science, PubMed, the Cochrane Library, Embase, and Clinical Trials. The search terms included "probiotics", "irritable bowel syndrome" and "child". Here, we use the PubMed database as an example (Fig 1).

### Study screening, data extraction and assessment of bias

Data were collected independently by two researchers. Studies that did not meet the inclusion or exclusion criteria were eliminated, and eligible studies were screened by reading the title, keywords, abstract and full text. Then, the research data were extracted and checked, and disagreements were discussed or a decision was made by the author. The studies were selected by Endnote X9 software. The extracted data included the following:

1. Basic information of the study, including title, author and year of publication;
2. Characteristics of the included study, consisting of study duration, sample size of test group and control group, and intervention measures;
3. Outcome indicators and data included;
4. Collection of risk of bias assessments. The risk of bias in the included studies was assessed by using the RCT bias risk assessment tool recommended in the Cochrane Handbook for Systematic Reviews of Interventions (5.1.0) [17].

### Statistical analysis

RevMan 5.4.1 and STATA 16 software were used for the meta-analysis. The dichotomous variables are expressed as relative risk (RR) as an effect indicator, the continuous variables are expressed as standard mean difference (SMD) and mean difference (MD) as effect indicators, and the estimated value and 95% confidence interval (CI) were included as effect analysis statistics. Since there are many types of probiotics, there might be differences between different probiotics. Therefore, regardless of whether I^2^ was higher than 50%, we adopted a random effects model. The significance level was set at α = 0.05.

## Results

### Retrieved results

**Fig 2:**
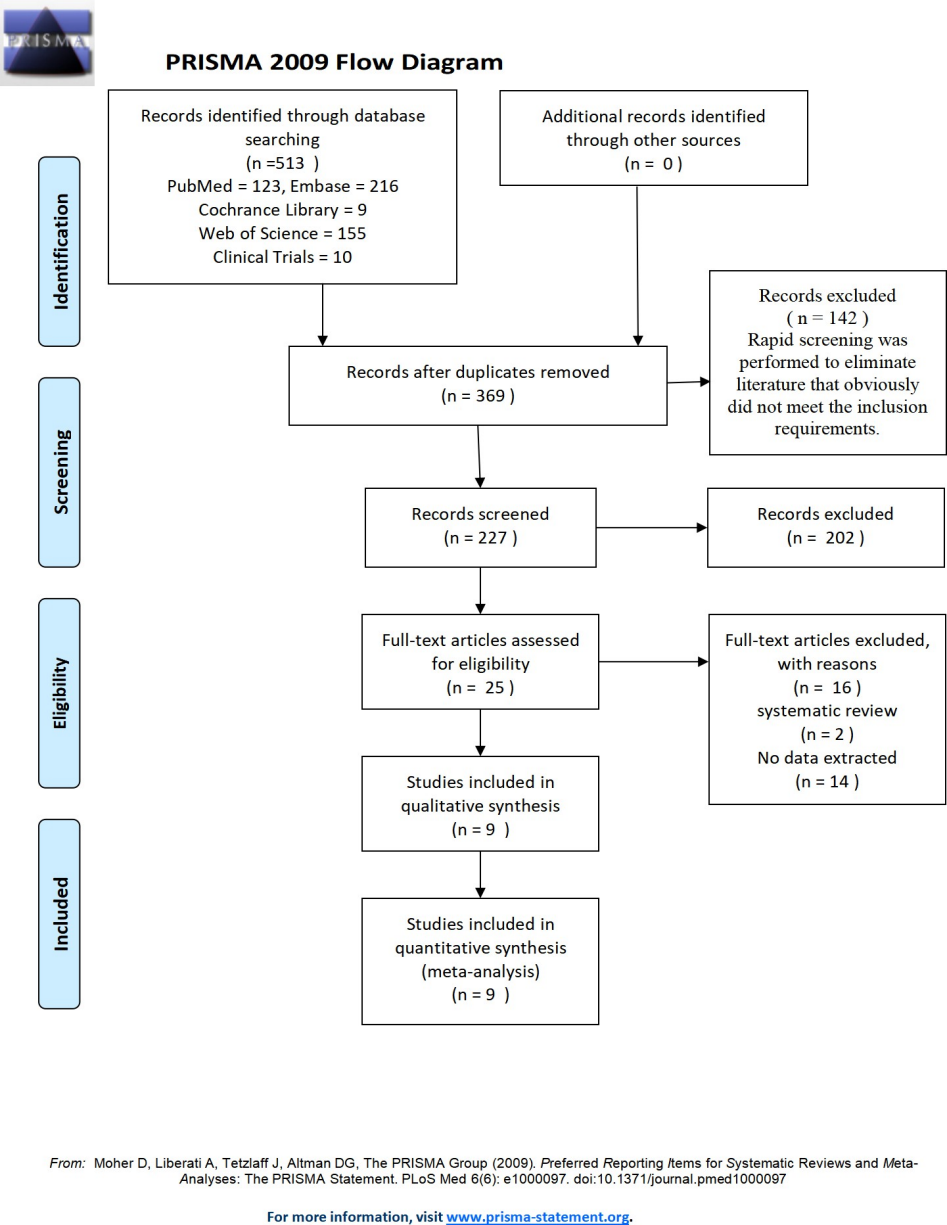
PRISMA flow diagram of evidence acquisition during the study.

A total of 440 studies were initially selected, and nine studies were finally included after screening. All of the included studies were written in English. The literature screening process and results are shown in Fig 2.

### Basic information of studies

The basic characteristics of the included studies and the risk of bias evaluation are shown in Table 1.

**Table 1:** Basic information and bias risk assessments of the study.

Author	Year	Country	Registration Number	Intervening measure(I)	Control measure(C)	Daily intake of colonies (10^9 CFU/day)	Sample size		Treatment duration	Diagnostic criteria	Outcome included	Criteria for abdominal pain score	Literature quality score						
							I	C					Random sequence generation	Allocation concealment	Blinding of participants and personnel	Blinding of outcome assessment	Incomplete outcome data	Selective reporting	Other bias
Başturk, A.	2016	Turkey	-	Synbiotic (Bifidobacterium lactis B94 with inulin)	Prebiotic (inulin)	10	23	24	4 week	Rome III	**⑥**	-	Low	Low	Low	Low	Low	Low	Unclear
Bauserman, M.	2005	US	-	Lactobacillus GG	Placebo	20	25	25	6 week	Rome II	**①④⑥**	Abdominal pain severity score	Low	Low	Low	Low	Unclear	Low	Unclear
Francavilla, R.	2010	Italy	NCT00876291	Lactobacillus GG	Placebo	6	42	38	8 week	Rome II	**①④⑤**	visual an alog scale and the Faces Pain Scale (FPS)	Low	Low	Low	Low	Low	Low	Unclear
Gawrońska, A.	2007	Poland	-	Lactobacillus GG	Placebo	6	18	19	4 week	Rome II	**①③④⑤**	Facial responses were transformed into a score that ranged from 0 (a relaxed face) to 6 (intense pain).	Low	Low	Low	Low	Low	Low	Unclear
Giannetti, E.	2017	Italy	NCT02566876	A probiotic mixture of Bifidobacterium infantis M-63, breve M-16V and longum BB536	Placebo	5	48	48	6 week	Rome III	**③**	-	Low	Low	Low	Low	Unclear	Low	Unclear
Guandalini, S.	2015	Italy	-	VSL#3	Placebo	225	59	59	6 week	Rome II	**①②**	subject’s global relief of symptoms (SGARC)	Unclear	Low	Low	Low	Unclear	Low	Unclear
Kianifar, Hamidreza	2015	Iran	IRCT201205219825N1	Lactobacillus GG	Placebo	20	26	26	4 week	Rome III	**①**	A five-point Likert scale	Unclear	Low	Low	Low	Unclear	Low	Unclear
Rahmani, P.	2020	Iran	-	Probiotic chewable tablets (containing 108 colony forming units L. reuteri)	Placebo	Unclear	15	15	4 week	Rome III	**①③⑤**	Wang-Baker FACES Pain Rating Scale (WBFPRS)2	Low	Low	Low	Low	Low	Low	Unclear
Sudha, M. Ratna	2018	India	CTRI/2017/02/007810	Bacillus coagulans Unique IS2	Placebo	2	72	69	8 week	Rome III	**①②**	0–10 Likert scale	Low	Low	Low	Low	Low	Unclear	Unclear

Outcomes: (1)Abdominal pain score, (2)Subject’s Global Assessment of Relief (SGARC) score, (3)Abdominal pain treatment success, (4)Abdominal pain relief, (5)Frequency of abdominal pain, (6)Rate of bloating after treatment.

### Meta-analysis results

Nine RCTs [18–26] were included in this study, and a total of 651 individuals were included. Among them, there were 328 people in the probiotics group and 323 people in the placebo group.

#### Abdominal pain score

**Fig 3:**
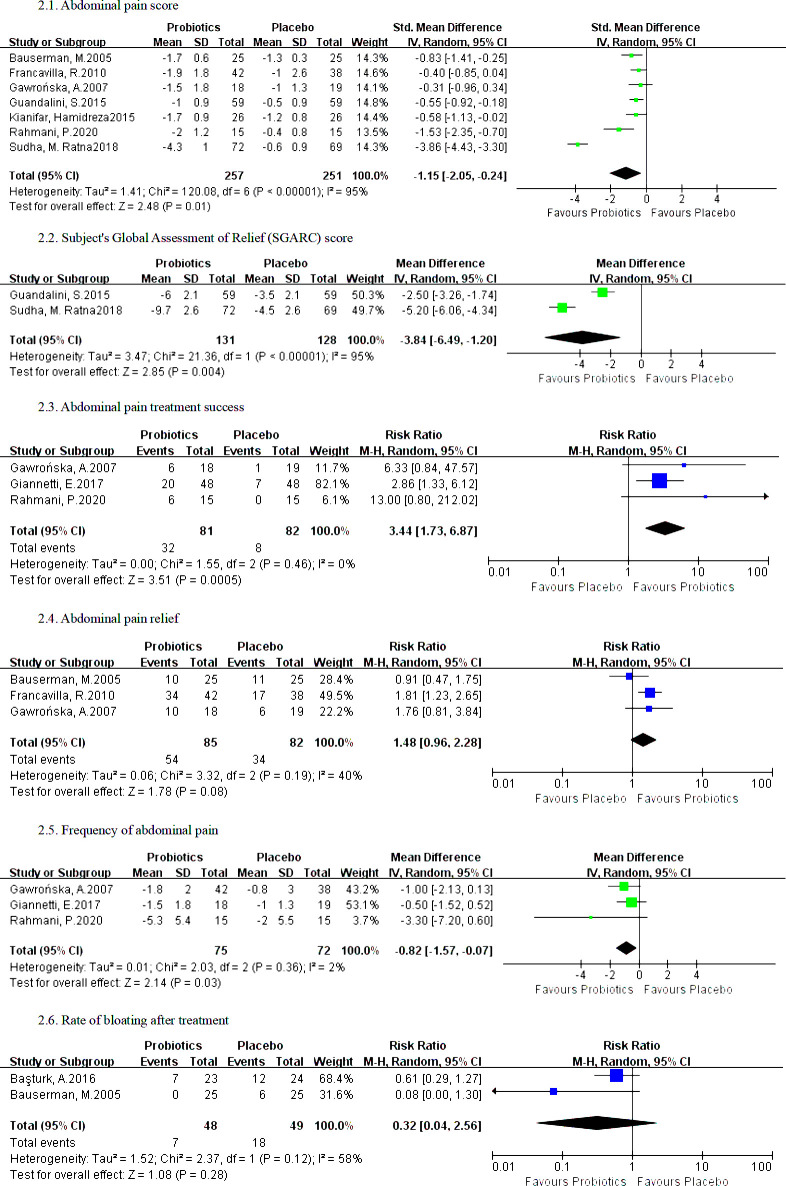
Forest plot comparing the probiotic and placebo groups.

Seven RCTs reported differences in abdominal pain scores between the probiotics group and the placebo group. A random effect model was adopted. Compared with placebo, probiotics could significantly reduce the abdominal pain score (I^2^ = 95%, SMD = -1.15, 95% (-2.05, -0.24), *P* = 0.01) (Fig 3).

#### SGARC score

Two RCTs reported differences in SGARC scores between the probiotic group and the placebo group. A random effect model was adopted. Compared with placebo, probiotics could significantly reduce the SGARC score (I^2^ = 95%, MD = -3.84, 95% (-6.49, -1.20), *P* = 0.004) (Fig 3).

#### Abdominal pain treatment success

Three RCTs reported differences in abdominal pain treatment success between the probiotics group and the placebo group. A random effect model was adopted. Compared with placebo, probiotics could significantly increase abdominal pain treatment success (I^2^ = 0%, RR = 3.44, 95% (1.73, 6.87), *P* = 0.0005) (Fig 3).

#### Abdominal pain relief

Three RCTs reported differences in abdominal pain relief between the probiotic group and the placebo group. A random effect model was adopted. Compared with the placebo, probiotics might have the potential to provide more abdominal pain relief, but the difference between groups was not statistically significant (I^2^ = 40%, RR = 1.48, 95% (0.96, 2.28), *P* = 0.08) (Fig 3).

#### Frequency of abdominal pain

Three RCTs reported differences in the frequency of abdominal pain between the probiotic group and the placebo group. A random effect model was adopted. Compared with placebo, probiotics could significantly decrease the frequency of abdominal pain (I^2^ = 2%, MD = -0.82, 95% (-1.57, -0.07), *P* = 0.03) (Fig 3).

#### Rate of bloating after treatment

Two RCTs reported differences in the rate of bloating after treatment between the probiotics group and the placebo group. A random effect model was adopted. In the comparison of bloating after treatment, the difference between probiotics and placebo was not statistically significant (I^2^ = 58%, RR = 0.32, 95% (0.04, 2.56), *P* = 0.28) (Fig 3).

#### Standard abdominal pain and daily intake of probiotics

**Fig 4:**
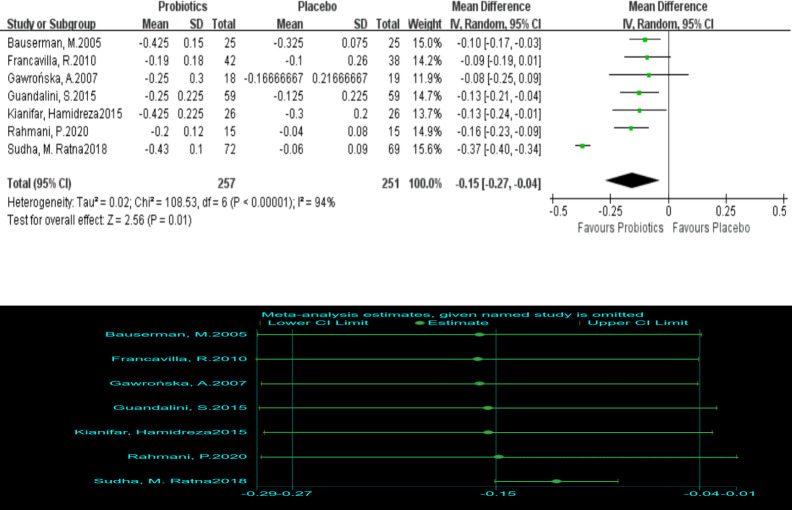
Forest and sensitivity analysis plot comparing SAP between the probiotic and placebo groups.

**Fig 5:**
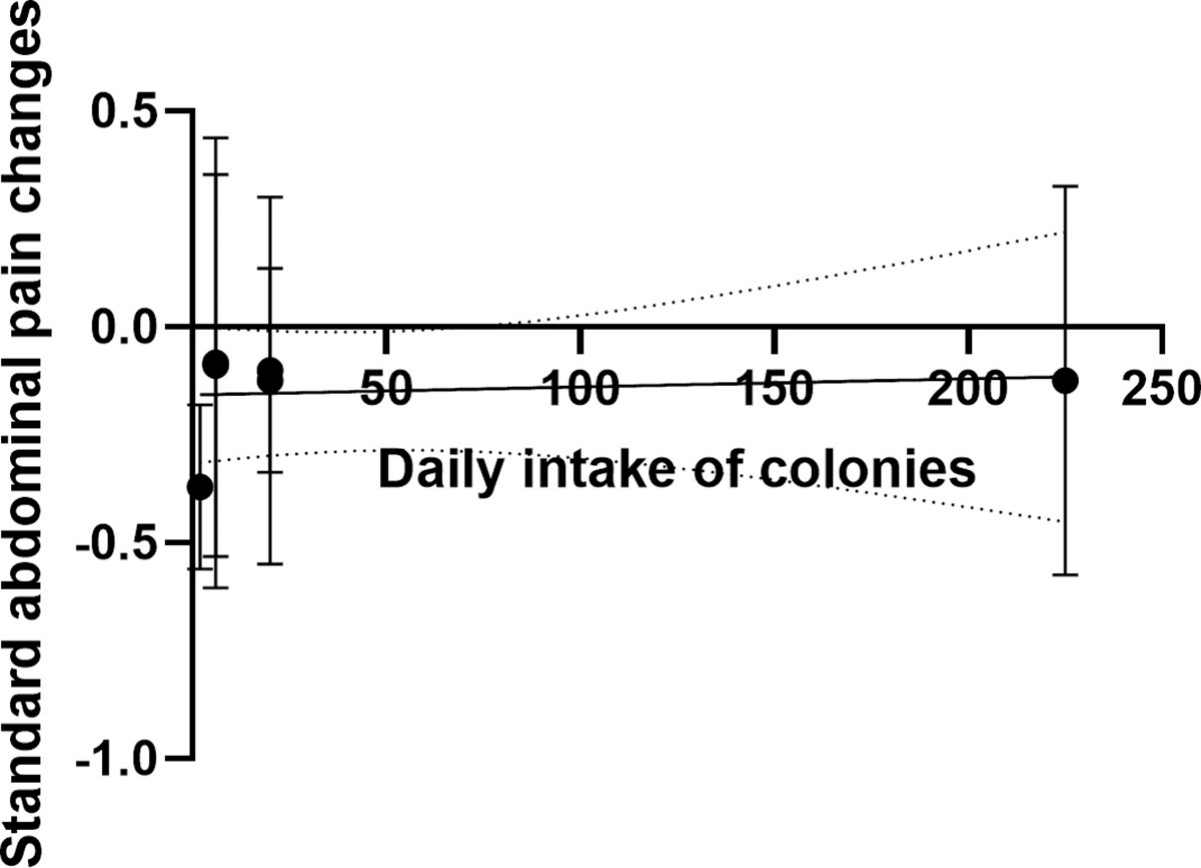
The relationship between daily intake of probiotics and SAP.

Six RCTs reported differences in SAP between the probiotic group and the placebo group. A random effect model was adopted. Compared with placebo, probiotics could significantly reduce SAP (I^2^ = 94%, MD = -0.15, 95% (-0.27, -0.04), P = 0.01). Sensitivity analysis found that Sudha’s research was the main source of heterogeneity, but the inclusion or deletion of his research did not change the results of this meta-analysis, so the result was reliable (Fig 4). Moreover, we compared SAP and daily intake of probiotics and found that the daily intake of probiotics is not significantly related to SAP. It might not be possible to reduce abdominal pain by increasing the daily intake of probiotics (Fig 5).

## Discussion

Since the outcomes of each RCT were different, the data synthesis of this meta-analysis was also scattered. There are many different measurement standards for the severity of abdominal pain (Table 1), so we used the SMD as the effect index, while other outcomes were mostly provided by only 2–3 RCTs. According to the results, probiotics have good efficacy in children with IBS with abdominal pain, and other outcomes tend to support this conclusion. However, increasing the daily intake of probiotics was not found to reduce abdominal pain to a greater extent. The evidence for the efficacy of probiotics in children with bloating is still insufficient.

IBS is a common functional gastrointestinal disorder. The etiology of IBS might be diverse. Possible causes of IBS include chronic or acute inflammation, chronic or acute infections, bile acid malabsorption, alterations in ion channels, disaccharidase deficiency, etc. These factors lead to the disorder of the normal gut microbiota and loss of the barrier function of the normal gut microbiota. Normal gut microbiota maintain the pH value of the intestine, nourish epithelial cells, and assist in digestion [27]. The absence of normal gut microbiota weakens the tight junctions of intestinal epithelial cells, facilitating the release of a series of inflammatory mediators that in turn results in abdominal pain and bloating [8,28].

Research by Bożena Cukrowska et al. suggested that the gut microbiota was the backbone of the integrity of the intestinal epithelial cells and immune homeostasis [29]. After studying animals lacking intestinal microbiota, it was concluded that the intestinal microbiota played an important role in the local mucosal gut-associated lymphoid tissue and the intestinal immune system [30,31]. For example, compared with conventionally cultured mice, the Peyer’s patches in mice lacking intestinal microbiota were underdeveloped, and the numbers of IgA-secreting plasma cells and lymphocytes were reduced. Animals lacking intestinal microbiota have increased levels of secretory immunoglobulin A due to colonizing intestinal microbes. Secretory immunoglobulin A is a natural antibody that in intestine that participates in the defense against a wide range of microorganisms and toxic molecules [32–34]. Similar study also showed that intestinal microbes could induce the recruitment and activation of intraepithelial lymphocytes, which could protect epithelial cells and strengthen their barrier function [35]. The study by Markus B Geuking suggested that the intestinal microbiota had an impact on the terminal differentiation of CD4+ Th cells [36].

According to the latest research by Daisuke Tokuhara [37], the intestinal microbiota is a key player in the development and regulation of the gut mucosal immune system. Dysbiosis of the intestinal microbiota promotes the development of non-alcoholic fatty liver disease, including disruption of the gut barrier, portal transport of bacterial endotoxin (lipopolysaccharide) to the liver, altered bile acid profiles, and decreased concentrations of short-chain fatty acids. Probiotics could improve intestinal microbiota. Probiotics enhance the barrier function of the gut, e.g., mucus layer, secretory IgA levels and tight junction tension, and improve the gut microbiota composition, bile acid homeostasis, and short-chain fatty acids production.

Probiotics are microorganisms that are beneficial to people [38,39]. Beneficial microorganisms in the human body include yeast, probiotic spores, *Clostridium butyricum*, and bacteria in the *Lactobacillus*, *Bifidobacterium*, and *Actinomycetes* genera etc. These microorganisms could promote the digestion and absorption of nutrients, reduce serum cholesterol levels [40], improve immunity [39], maintain the balance of the gut microbiota, increase antioxidant levels [41], inhibit intestinal inflammation [42], and protect the intestinal mucosal barrier [43]. Francesca Algieri et al. showed that probiotics had intestinal anti-inflammatory effects, but those effects differed slightly with regard to the expression of miRNAs [44]. The study by Haiyan Xu showed that probiotics were not equally effective in all trial participant. Furthermore, the initial composition of the intestinal flora might affect the clinical efficacy of probiotic treatment, and the pretreatment analysis of the intestinal microbiota might support the personalization of the probiotic program to optimize the treatment effect [45].

This study indicated that simply increasing the daily intake of probiotics did not significantly improve abdominal pain in IBS patients. There is still no reliable evidence regarding whether the combination of different probiotics could improve abdominal pain. Different probiotics have different functions in humans. The effects of different combinations of probiotics might also be different [46]. Hundreds of probiotics and thousands of combinations exist and need further investigation.

With regard to studies on the efficacy of probiotics on IBS, most of the research subjects have been adults [47], and there have been few studies conducted with children. Whether there is a difference between the mechanism and the efficacy of probiotics in adults and children is still inconclusive. More high-quality trials are needed to verify the efficacy of probiotics in children with IBS.

### Limitations of this meta-analysis

1. The outcome indicators were too scattered, which was not conducive to the synthesis of the effect size.
2. The diversity of probiotics affected the handling of heterogeneity.
3. There is a lack of uniform standards for the assessment of the severity of IBS in children.

## Conclusions

Probiotics are effective at treating abdominal pain caused by IBS in children; however, there was not a significant correlation between abdominal pain and the amount of probiotics ingested. Choosing the most suitable probiotics may be relatively more important. More attention should be given to IBS in children, and a unified evaluation standard should be adopted.

## Supporting information

S1 Checklist PRISMA 2009 checklist. (DOC)
